# Physiological, Metabolome and Gene Expression Analyses Reveal the Accumulation and Biosynthesis Pathways of Soluble Sugars and Amino Acids in Sweet Sorghum under Osmotic Stresses

**DOI:** 10.3390/ijms25168942

**Published:** 2024-08-16

**Authors:** Yan-Nong Cui, Shi-Jie Yan, Yi-Nuo Zhang, Rong Wang, Le-Ling Song, Yue Ma, Huan Guo, Pei-Zhi Yang

**Affiliations:** College of Grassland Agriculture, Northwest A&F University, Yangling 712100, China; cuiyn@nwafu.edu.cn (Y.-N.C.); yjay@nwafu.edu.cn (S.-J.Y.); yinuo_zhang2003@163.com (Y.-N.Z.); wr00142023@163.com (R.W.); songleling1@163.com (L.-L.S.); m17319936758@163.com (Y.M.)

**Keywords:** drought, soluble sugars, amino acids, osmotic adjustment, C_4_ crop

## Abstract

Water scarcity is a major environmental constraint on plant growth in arid regions. Soluble sugars and amino acids are essential osmolytes for plants to cope with osmotic stresses. Sweet sorghum is an important bioenergy crop and forage with strong adaptabilities to adverse environments; however, the accumulation pattern and biosynthesis basis of soluble sugars and amino acids in this species under osmotic stresses remain elusive. Here, we investigated the physiological responses of a sweet sorghum cultivar to PEG-induced osmotic stresses, analyzed differentially accumulated soluble sugars and amino acids after 20% PEG treatment using metabolome profiling, and identified key genes involved in the biosynthesis pathways of soluble sugars and amino acids using transcriptome sequencing. The results showed that the growth and photosynthesis of sweet sorghum seedlings were significantly inhibited by more than 20% PEG. After PEG treatments, the leaf osmotic adjustment ability was strengthened, while the contents of major inorganic osmolytes, including K^+^ and NO_3_^−^, remained stable. After 20% PEG treatment, a total of 119 and 188 differentially accumulated metabolites were identified in the stems and leaves, respectively, and the accumulations of soluble sugars such as raffinose, trehalose, glucose, sucrose, and melibiose, as well as amino acids such as proline, leucine, valine, serine, and arginine were significantly increased, suggesting that these metabolites should play key roles in osmotic adjustment of sweet sorghum. The transcriptome sequencing identified 1711 and 4978 DEGs in the stems, as well as 2061 and 6596 DEGs in the leaves after 20% PEG treatment for 6 and 48 h, respectively, among which the expressions of genes involved in biosynthesis pathways of sucrose (such as *SUS1*, *SUS2*, etc.), trehalose (including *TPS6*), raffinose (such as *RAFS2* and *GOLS2*, etc.), proline (such as *P5CS2* and *P5CR*), leucine and valine (including *BCAT2*), and arginine (such as *ASS* and *ASL*) were significantly upregulated. These genes should be responsible for the large accumulation of soluble sugars and amino acids under osmotic stresses. This study deepens our understanding of the important roles of individual soluble sugars and amino acids in the adaptation of sweet sorghum to water scarcity.

## 1. Introduction

The low precipitation and intense evaporation in arid regions primarily limit soil water availability to roots and impose osmotic stress on plants [1,2]. Traditional crops grown in these areas require relatively high irrigations but are not capable of achieving adequate productions [3]. Sweet sorghum [*Sorghum bicolor* (L.) Moench], a C_4_ plant within the Poaceae family, is a valuable bioenergy crop because it contains extremely high fermentable sugars, and its stalks and leaves are directly used as resources for sugar and ethanol production; additionally, this species also serves as a forage due to its prominent palatability and abundant dietary nutrient contents [4,5]. In contrast to traditional cereal crops, sweet sorghum possesses strong tolerances to environmental stresses including drought and salinity, which enables it to thrive in marginal lands [3]. Therefore, understanding the adaptative mechanisms of sweet sorghum to environmental stresses and exploring gene resources for the development of stress-resistant genotypes of traditional crops are high priorities for promoting agricultural productivity and ecological restoration in arid regions [6,7,8].

There are diverse adaptative strategies evolved by higher plants to cope with drought or osmotic stress. These include expanding root systems to absorb more soil water, closing stomatal pores or reducing leaf areas to diminish transpirational water loss, accumulating cuticular wax on the leaf surface to restrict none-stomatal water loss, and absorbing inorganic ions or biosynthesizing soluble organic metabolites to enhance the osmotic adjustment (OA) ability [9,10,11]. OA is the most common strategy of plants to enable water influx into cells and maintain the tissue hydration status, and the large accumulation of organic osmolytes has been considered a general protective mechanism of plants under water deficiency conditions [12,13]. Soluble sugars (mainly small molecular carbohydrates including monosaccharide, disaccharide, and trisaccharide) are indispensable organic osmolytes for higher plants [14]. It has been extensively reported that the contents of total soluble sugars in many plants are greatly increased under drought and osmotic stresses [15,16,17]. Actually, the accumulating patterns of individual soluble sugar, including sucrose, raffinose, and galactinol, in the leaves of three *Craterostigma* species are distinct under drought stresses; additionally, *Codonopsis pilosula* preferentially accumulates higher quantities of trehalose, raffinose, and maltotetraose rather than other soluble sugars under drought stresses [18,19]. Therefore, the role of individual sugar in drought resistance among plant species might be different. In addition, several members of soluble sugars are also involved in other aspects to improve the stress tolerances of plants. For instance, trehalose, raffinose, fructose, and sucrose are signal molecules and can activate the immune system of plants; raffinose and galactinol act as non-enzymatic antioxidants for the degradation of reactive oxygen species (ROS) [19,20,21]. Although the contents of sucrose, glucose, and fructose in sweet sorghum under drought conditions have been analyzed [6,22], the accumulating patterns of other soluble sugars in this species under conditions of water scarcity have not been reported.

Except for soluble sugars, free amino acids are also considered as organic osmolytes for plants [23]. The contents of proline in many plant species have been found to largely increase under abiotic stresses. Proline also plays an important role in ROS degradation and regulating cell redox status [24,25]. In fact, some other members of amino acids are also positively associated with the adaptation of plants to drought or osmotic stress; for example, the contents of asparagine, phenylalanine, methionine, and serine in hybrid bermudagrass are significantly increased after drought treatments; the fold increase in branched-chain amino acids (BCAAs; leucine, isoleucine, and valine) is higher than that of proline in some plant species under drought stresses [26,27]. These BCAAs are thought to act as compatible osmolytes and alternative energy resources to enhance the drought resistance of plants [28]. Although the accumulation of proline in sweet sorghum under drought stresses has been investigated [29], the function of other amino acids in this species in adapting to water scarcity still remains elusive. In addition, unlike inorganic osmolytes such as K^+^ and NO_3_^−^ that are absorbed by plant roots, organic osmolytes are biosynthesized through complicated metabolic processes and regulated by many enzymes [30,31]. It has been reported that the expressions of genes encoding key enzymes in the biosynthesis processes of certain metabolites such as raffinose and BCAAs are substantially induced by drought stresses to enhance their accumulations in plant tissues [20,26]. Nevertheless, the molecular basis underlying the biosynthesis and metabolism of sugars and amino acids in sweet sorghum under conditions of water scarcity is still unclear.

The integrated metabolome and transcriptome analysis is widely used in uncovering plants’ responses to environmental stresses. The metabolome profiling can analyze the accumulation changes in metabolites among different treatment conditions, and transcriptome sequencing can provide insights into the molecular processes by monitoring gene expressions after treatments [32,33]. In this study, in order to reveal the accumulation changes in soluble sugars and amino acids in sweet sorghum under osmotic stresses, and preliminarily investigate the molecular basis underlying the biosynthesis of these metabolites, we first evaluated the growth, photosynthesis, tissue inorganic osmolyte contents and leaf osmotic adjustment capacity in sweet sorghum under a series of PEG6000-induced osmotic stresses. Next, we analyzed differentially accumulated sugars and amino acids in the stems and leaves after 20% PEG treatment using metabolome profiling. Finally, we identified key genes involved in the biosynthesis processes of sugars and amino acids under osmotic stress using transcriptome sequencing and the qRT-PCR method.

## 2. Results

### 2.1. The Effects of Osmotic Stresses on the Growth and Photosynthesis of Sweet Sorghum

**Figure 1:**
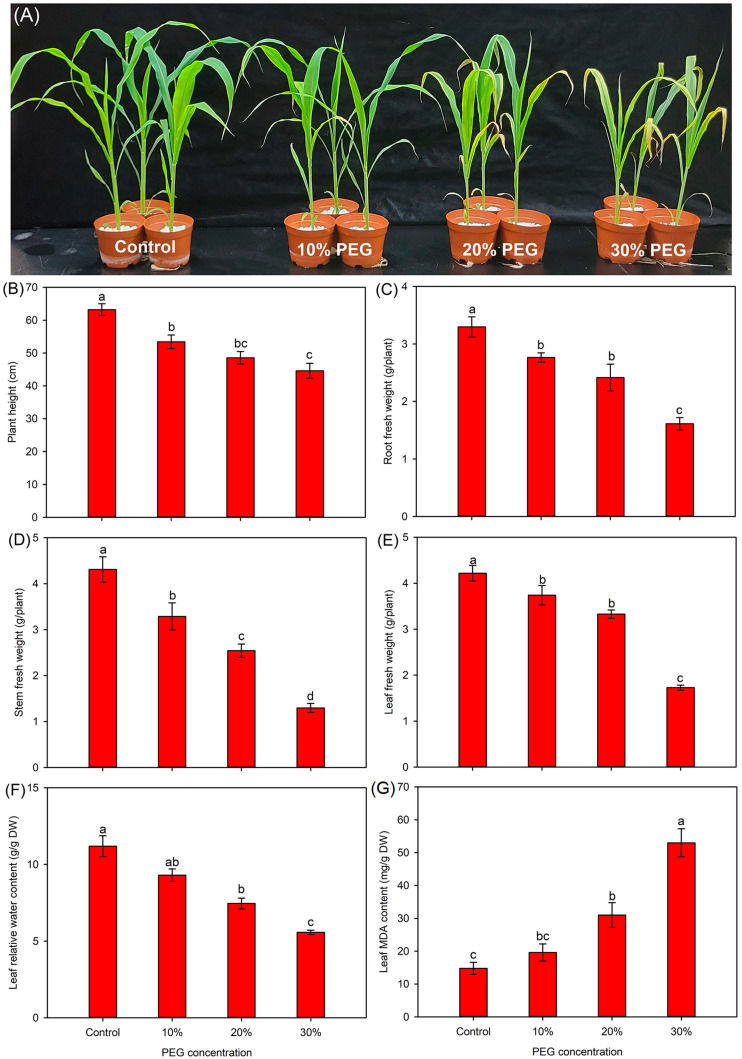
Effects of 10–30% PEG treatments on the growth-related parameters of sweet sorghum. (**A**) growth photograph; (**B**) plant height; (**C**) root fresh weight; (**D**) stem fresh weight; (**E**) leaf fresh weight; (**F**) leaf relative water content; (**G**) leaf MDA content. Data are means (±SDs), *n* = 6. Different letters indicate significant differences determined by Tukey’s HSD test (*p* < 0.05).

As shown in Figure 1 and Figure S1, compared with the control, 10% PEG had no significant effect on the tissue dry weight (DW), leaf relative water content (RWC), and leaf malonaldehyde (MDA) content of sweet sorghum seedlings, while it significantly decreased the plant height (PH) and tissue fresh weight (FW). Seedlings treated with 20% PEG initially showed a visually wilting symptom in the leaf tips; the PH, tissue FW and DW, and leaf RWC under this treatment were significantly decreased compared to those under the control conditions (Figure 1 and Figure S1). After 30% PEG treatment, the leaves of seedlings were severely impaired, and all growth-related parameters were significantly reduced, accompanied by a drastically increased leaf MDA content (Figure 1 and Figure S1).

**Figure 2:**
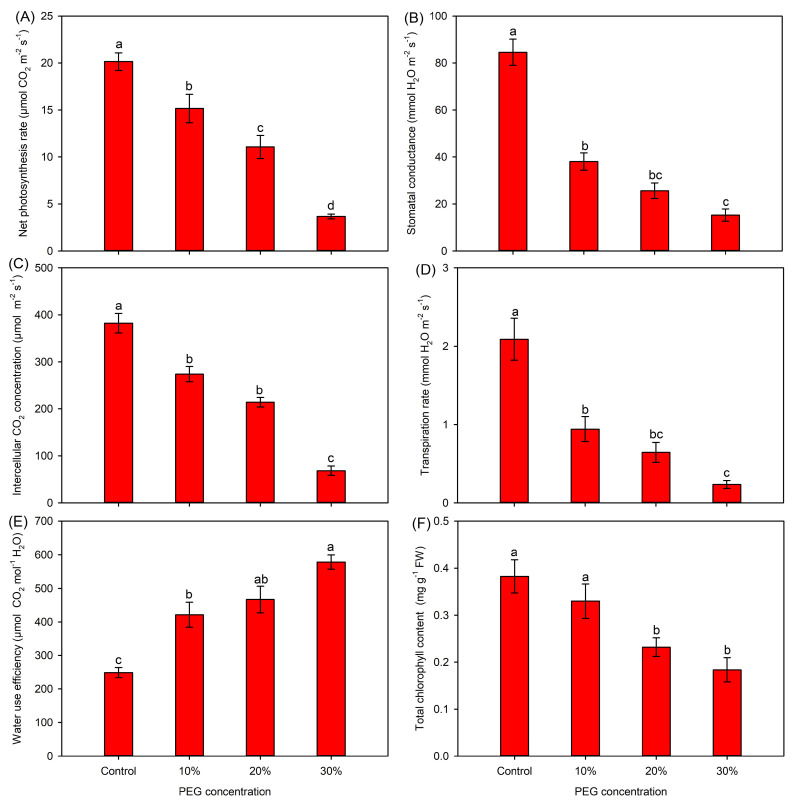
Effects of 10–30% PEG treatments on the photosynthesis-related parameters of sweet sorghum. (**A**) Net photosynthesis rate; (**B**) stomatal conductance; (**C**) intercellular CO_2_ concentration; (**D**) transpiration rate; (**E**) water-use efficiency; (**F**) total chlorophyll content. Data are means (±SDs), *n* = 6. Different letters indicate significant differences determined by Tukey’s HSD test (*p* < 0.05).

Compared with the control, all gas-exchange parameters showed significantly decreasing trends under PEG treatments (Figure 2A–D). Especially, the Gs and Tr were drastically decreased by more than 50% under PEG treatments (Figure 2B,D). In contrast, PEG treatments significantly increased the leaf water-use efficiency (Figure 2E). In comparison with the control, the 10% PEG treatment did not affect the total chlorophyll content, while 20% and 30% PEG treatments significantly decreased this parameter (Figure 2F).

### 2.2. The Effects of Osmotic Stresses on the Tissue K^+^ and NO_3_^−^ Contents and Leaf Osmotic Adjustment in Sweet Sorghum

**Figure 3:**
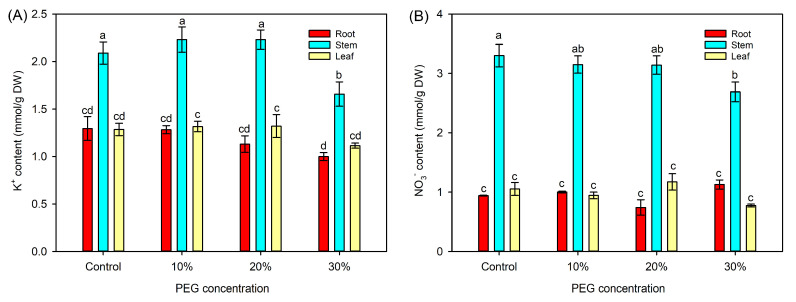
Effects of 10%–30% PEG treatments on the tissue K^+^ and NO_3_^−^ contents in sweet sorghum. (**A**) tissue K^+^ content; (**B**) tissue NO_3_^−^ content. Data are means (±SDs), *n* = 6. Different letters indicate significant differences determined by Tukey’s HSD test (*p* < 0.05).

In comparison with the control, the 10% and 20% PEG treatments did not affect the tissue K^+^ and NO_3_^−^ contents, while 30% PEG treatment significantly decreased the contents of both ions in the stems (Figure 3). The leaf water potential (Ψ_w_) and osmotic potential (Ψ_s_) were significantly decreased under all PEG treatments, with the lowest values observed under 30% PEG treatment. In addition, the leaf turgor pressure (Ψ_t_) under 20% and 30% PEG treatments was also significantly reduced (Table 1).

### 2.3. Metabolome Profiling of Sweet Sorghum under Osmotic Stresses

**Figure 4:**
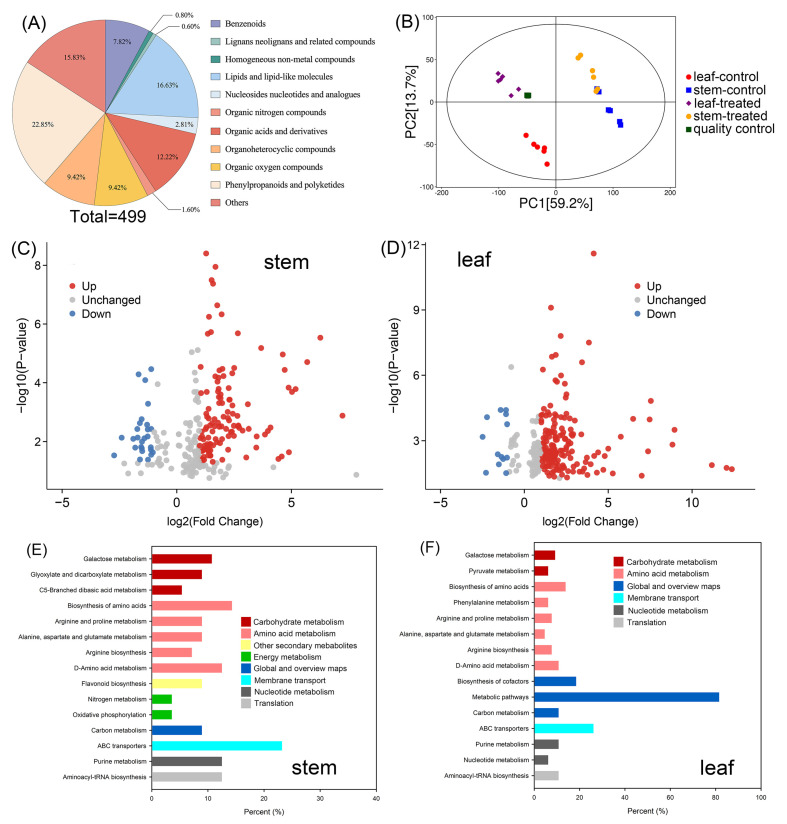
Metabolic analysis of sweet sorghum under 20% PEG treatment. (**A**) The classification of all 499 metabolites identified by metabolome profiling; (**B**) principal component analysis of metabolites in each group; leaf-control and stem-control refer to the leaf and stem samples collected from seedlings irrigated with Hoagland solution, respectively, and leaf-treated and stem-treated refer to the leaf and stem samples collected from seedlings treated with PEG, respectively; (**C**,**D**) the volcano plot of increasing accumulated (red dots), unchanged (gray dots) and decreasing accumulated (blue dots) metabolites in the stems and leaves, respectively; (**E**,**F**) KEGG analysis of DAMs in the stems and leaves, respectively.

In total, 499 metabolites in the stems and leaves of sweet sorghum were identified after metabolome profiling (Figure 4A and Table S1). The principal component analysis (PCA) showed that the scores of PC1 and PC2 in the four quality controls were highly coincident (Figure 4B). The scores of PC1 and PC2 of the six replicates in each treatment group (leaf-control, stem-control, leaf-treated, and stem-treated, respectively) had a high similarity (Figure 4B). The differences in the PCA model between PEG-treated leaf samples and control leaf samples were more obvious than those between PEG-treated stem samples and control stem samples (Figure 4B), suggesting that PEG treatment had a more pronounced effect on the metabolic processes in the leaves than those in the stems.

After 20% PEG treatment for 5 d, a total of 146 (119 upregulated, 27 downregulated) differentially accumulated metabolites (DAMs) were identified in the stems, and 188 (175 upregulated, 13 downregulated) DAMs were identified in the leaves (Figure 4C,D, Tables S2 and S3). KEGG analysis showed that, among these DAMs, many members were enriched into the carbohydrate metabolism pathways, including the galactose metabolism, glyoxylate, and dicarboxylate metabolism, and C5-branched dibasic acid metabolism, or enriched into the amino acid metabolism pathways, including the arginine and proline metabolism, alanine, aspartate and glutamate metabolism, arginine biosynthesis, as well as D-amino acid metabolism (Figure 4E,F).

### 2.4. The Identification of DAMs Categorized into Soluble Sugars and Amino Acids in the Stems and Leaves of Sweet Sorghum under Osmotic Stresses

**Figure 5:**
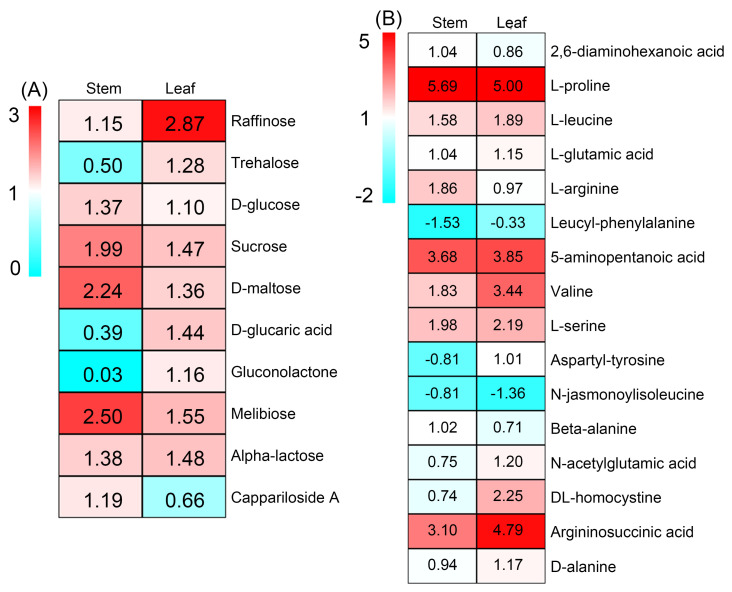
Heat maps showing the accumulation changes in metabolites categorized into (**A**) soluble sugars and (**B**) amino acids in the stems and leaves of sweet sorghum after 20% PEG treatment. The number in each block refers to the fold change in each metabolite.

There were 10 DAMs categorized into soluble sugars after 20% PEG treatment, among which the accumulations of raffinose, glucose, D-sucrose, D-maltose, melibiose and alpha-lactose were increased in both stems and leaves, the accumulations of trehalose, D-glucaric acid and gluconolactone were increased exclusively in the leaves, while the accumulation of cappariloside A was increased exclusively in the stems (Figure 5A). In total, 16 DAMs categorized into amino acids were detected after PEG treatment, among which seven members (L-proline, L-leucine, L-glutamic acid, 5-aminopentanoic acid, valine, L-serine, and argininosuccinic acid) displayed increasing accumulations in both stems and leaves; three members (2,6-diaminohexanoic acid, L-arginine, beta-alanine) displayed increasing accumulations exclusively in the stems; and four members (aspartyl-tyrosine, N-acetylglutamic acid, and DL-homocystine) showed increasing accumulations exclusively in the leaves (Figure 5B). The fold increases of L-proline, 5-aminopentanoic acid and argininosuccinic acid in both tissues after PEG treatment were >3 (Figure 5B).

### 2.5. Transcriptome Sequencing of Sweet Sorghum under Osmotic Stresses

After transcriptome sequencing, more than 70,005,000 raw reads were generated from 24 RNA-sequencing libraries in the stem and leaf samples of sweet sorghum (Table 2). By removing the adapter sequences and low-quality reads, approximately 70 million clean reads were obtained from each library, with clean bases > 10 billion, Q20 and Q30 rates > 98% and 94%, respectively, and GC contents > 55% (Table 2). In total, >94% of clean reads had a unique map with the reference genome sequence (Table S4). A total of 18632 new transcripts were identified, and 79% of the members were functionally annotated by aligning against the databases, including KEGG, Pathway, Nr, Uniprot, GO, KOG, Pfam, and TF (Table S5).

**Figure 6:**
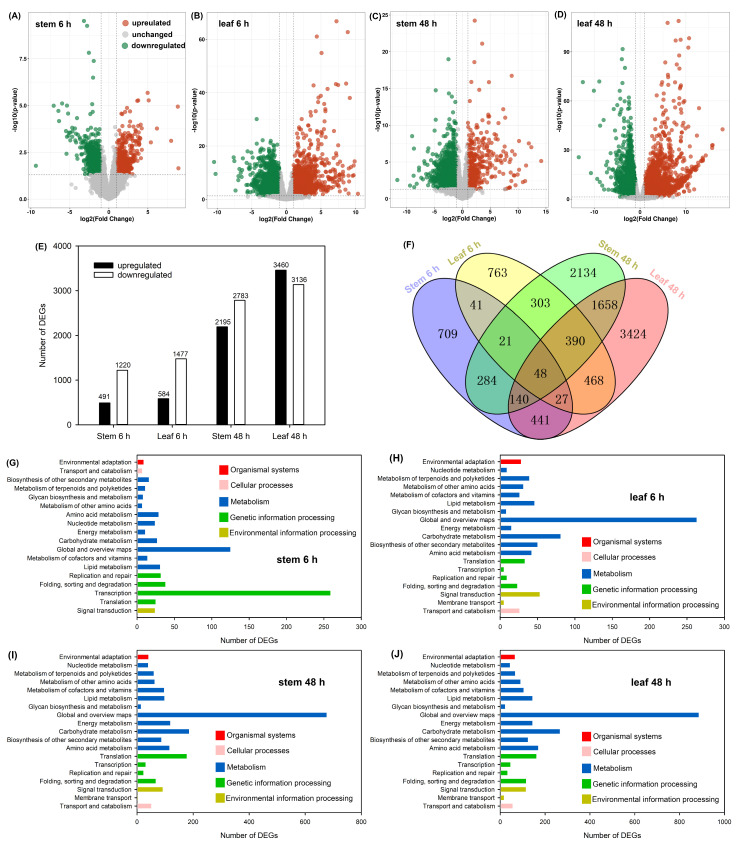
Analysis of DEGs in sweet sorghum after 20% PEG treatment. (**A**–**D**) the volcano plot of upregulated (red dots), unchanged (gray dots), and downregulated (green dots) DEGs in the stems and leaves after PEG treatment for 6 and 48 h, respectively; (**E**) the number of upregulated and downregulated DEGs; (**F**) the Venn diagram of DEGs; (**G**–**J**) KEGG analysis of DEGs in the stems and leaves after PEG treatment for 6 and 48 h, respectively.

Then, we analyzed the expression change in each unigene in the stems and leaves after 20% PEG treatment for 6 and 48 h, respectively. By using the threshold of |log_2_fold change| ≥ 1 and *p* < 0.05, the differentially expressed genes (DEGs) were identified (Figure 6A–D). A total of 1711 and 2061 DEGs were identified in the stems and leaves after 6 h of PEG treatment, respectively, and the majority of these DEGs were downregulated; when the treatment time prolonged to 48 h, a total of 4978 and 6596 DEGs were identified in the stems and leaves, respectively, among which 2195 and 3460 members were upregulated (Figure 6E). A Venn diagram showed that 48 DEGs were detectable in both stems and leaves after PEG treatment for both 6 and 48 h (Figure 6F). KEGG analysis indicated that many members of DEGs in the stems and leaves were enriched into metabolism pathways including the carbohydrate and amino acid metabolisms (Figure 6G–J).

In addition, there was a high correlation (R^2^ > 0.88) between gene expressions monitored by transcriptome sequencing and the qRT-PCR method (Figure S2), indicating the transcriptome data are reliable.

### 2.6. The Identification of DEGs Involved in the Biosynthesis Pathways of Soluble Sugars in Sweet Sorghum under Osmotic Stresses

**Figure 7:**
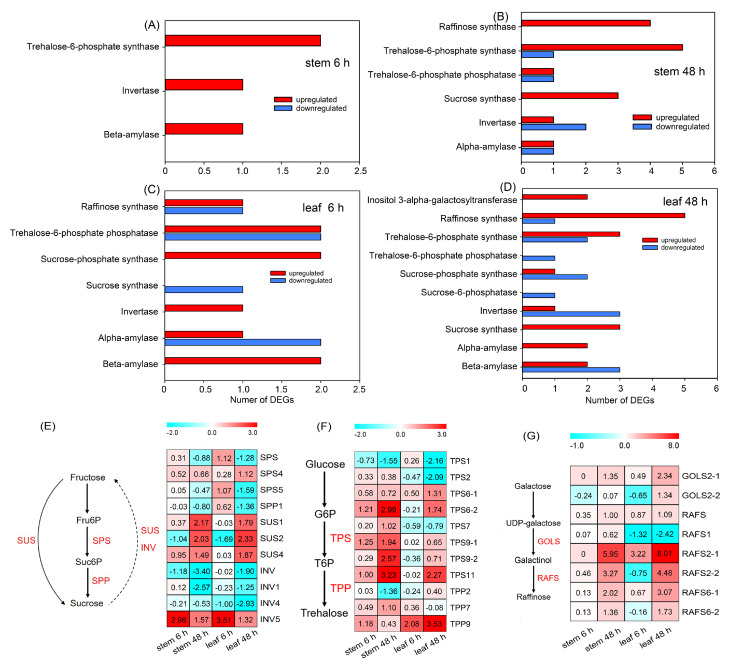
The analysis of DEGs related to the sugar metabolism in sweet sorghum after 20% PEG treatment. (**A**,**B**) The number of DEGs identified in the stems after PEG treatment for 6 and 48 h, respectively; (**C**,**D**) the number of DEGs identified in the leaves after PEG treatment for 6 and 48 h, respectively; (**E**) the expression level of genes related to sucrose metabolism; (**F**) the expression level of genes related to trehalose biosynthesis; (**G**) the expression level of genes related to raffinose biosynthesis. The number in each block refers to the fold change in each gene. Fru6P, fructose 6-phosphate; Suc6P, sucrose-6-phosphate; SPS, sucrose-phosphate synthase; SPP, sucrose-6-phosphatase; SUS, sucrose synthase; INV, invertase; G6P, glucose-6-phosphate; T6P, trehalose-6-phosphate; TPS, trehalose-6-phosphate synthase; TPP, trehalose-6-phosphate phosphatase; GOLS, galactinol synthase; RAFS, raffinose synthase.

As shown in Figure 7A and Table S6, four upregulated DEGs related to sugar metabolisms were identified in the stems after PEG treatment for 6 h, including two transcripts of trehalose-6-phosphate synthase (TPS, involved in trehalose biosynthesis [34]), one transcript of invertase (INV, involved in sucrose degradation [35]) and one transcript of beta-amylase (involved in starch degradation [36]). When the treatment time was prolonged to 48 h, the upregulated DEGs increased to 15 in the stems, including four transcripts of raffinose synthase (RAFS, involved in raffinose biosynthesis [20]), five transcripts of TPS and one transcript of trehalose-6-phosphate phosphatase (TPP, involved in trehalose biosynthesis [34]), three transcripts of sucrose synthase (SUS, involved in sucrose metabolism [30]), one transcript of INV and one transcript of alpha-amylase (involved in starch degradation [36]) (Figure 7B, Table S7). There were 15 and 32 DEGs related to the sugar metabolism in the leaves after PEG treatment for 6 and 48 h, respectively (Figure 7C,D, Tables S8 and S9). Several DEGs, such as transcripts of sucrose-phosphate synthase and sucrose-6-phosphatase (SPS and SPP, respectively, both involved in sucrose biosynthesis [30]), as well as galactinol synthase (GOLS, involved in raffinose biosynthesis [20]), were exclusively detectable in the leaves (Figure 7C,D, Tables S8 and S9).

The biosynthesis of sucrose in plants is catalyzed by SPS, SPP and SUS, and its degradation is catalyzed by SUS and INV [30]. Our results showed that the expressions of *SPS* and *SPP* mainly remained unchanged or were significantly downregulated in the stems of sweet sorghum after PEG treatment (Figure 7E). Differently, *SUS1*, *SUS2* and *SUS4* in both stems and leaves were significantly upregulated after 48 h of treatment (Figure 7E). After PEG treatment, three *INVs* showed significantly downregulating expressions, only *INV5* exhibited an upregulating expression (Figure 7E). TPS and TPP synergistically catalyze the biosynthesis of trehalose [34]. We identified 11 differentially expressed *TPS* and *TPP* after PEG treatment, among which the fold increases of *TPS6-2*, *TPS9-2*, and *TPS11* in the stems, as well as *TPS11* and *TPP9* in the leaves after 48 h of treatment were >2 (Figure 7F). The biosynthesis of raffinose is mainly catalyzed by GOLS and RAFS [20]. It was found that, after PEG treatment for both 6 and 48 h, *GOLS2-1* in the stems and leaves of sweet sorghum was significantly upregulated; several upregulated *RAFS* such as *RAFS2-1*, *RAFS2-2*, *RAF6-1*, and *RAFS6-2* in the stems and leaves were also identified, among which the fold increase of *RAFS2-1* was >5 (Figure 7G).

### 2.7. The Identification of DEGs Involved in the Biosynthesis Pathways of Amino Acids in Sweet Sorghum under Osmotic Stresses

**Figure 8:**
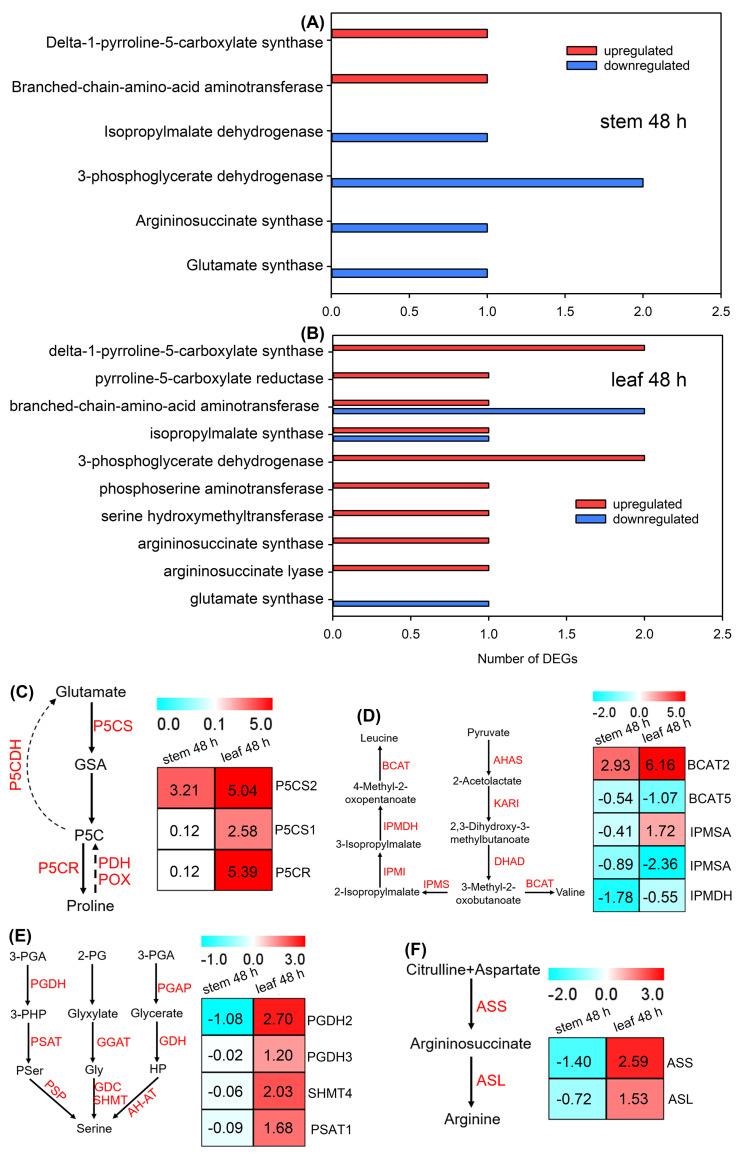
The analysis of DEGs related to the amino acid metabolism in sweet sorghum after 20% PEG treatment. (**A**,**B**) The number of DEGs identified in the stems and leaves after PEG treatment for 48 h, respectively; (**C**) the expression level of genes related to proline metabolism; (**D**) the expression level of genes related to leucine and valine biosynthesis; (**E**) the expression level of genes related to serine biosynthesis; (**F**) the expression level of genes related to arginine biosynthesis. The number in each block refers to the fold changes in each metabolite. GSA, glutamate-semialdehyde; P5C, pyrroline-5-carboxylic acid; P5CS, delta-1-pyrroline-5-carboxylate synthase; P5CR, pyrroline-5-carboxylate reductase; PDH, proline dehydrogenase; POX, proline oxidase; P5CDH, pyrroline-5-carboxylic acid dehydrogenase; AHAS, acetohydroxyacid synthase; KARI, ketolacid reducto-isomerase; DHAD, dihydroxyacid dehydratase; BCAT, branched-chain aminotransferase; IPMS, isopropylmalate synthase; IPMI, isopropylmalate isomerase; IPMDH, isopropylmalate dehydrogenase; 3-PGA, 3-phosphoglycerate; 2-PG, 2-phosphoglycolate; PGDH, phosphoglycerate dehydrogenase; PGAP, 3-PGA phosphatase; PSAT, phosphoserine aminotransferase; GGAT, glyoxylate glutamate aminotransferase; GDH, glycerate dehydrogenase; PSP, phosphoserine phosphatase; GDC, glycine decarboxylase complex; SHMT, serine hydroxymethyltransferase; AH-AT, alanine-HP aminotransferase; ASS, argininosuccinate synthase; ASL, argininosuccinate lyase.

As almost no DEGs related to amino acid biosynthesis were identified after PEG treatment for 6 h (data not shown), we presented DEGs in the stems and leaves of sweet sorghum after PEG treatment for 48 h. In total, seven DEGs encoding enzymes in the biosynthesis pathways of amino acids were identified in the stems, among which only one transcript of delta-1-pyrroline-5-carboxylate synthase (P5CS, involved in proline biosynthesis [37]), and one transcript of branched-chain aminotransferase (BCAT, involved in the biosynthesis of leucine and valine [26]) were upregulated (Figure 8A and Table S10). In leaves, the majority of identified DEGs were upregulated, including the transcripts of P5CS, pyrroline-5-carboxylate reductase (P5CR, involved in proline biosynthesis [37]), BCAT, isopropylmalate synthase (IPMSA, involved in the biosynthesis of leucine and valine [38]), phosphoglycerate dehydrogenase (PGHD), serine hydroxymethyltransferase (SHMT) and phosphoserine aminotransferase (PSAT) (all involved in serine biosynthesis [39]), argininosuccinate synthase and argininosuccinate lyase (ASS and ASL, respectively, both involved in arginine biosynthesis [40]) (Figure 8B and Table S11). 

The biosynthesis of proline is catalyzed by P5CS and P5CR, and it is degraded by proline dehydrogenase (PDH), proline oxidase (POX) and pyrroline-5-carboxylic acid dehydrogenase (P5CDH) [14]. In this study, no DEG related to proline degradation was detected in sweet sorghum after PEG treatment. Instead, three upregulated genes (*P5CS1*, *P5CS2* and *P5CR*) involved in proline biosynthesis were identified; notably, the fold increases of *P5CS2* and *P5CR* in the leaves after 48 h of PEG treatment were > 5 (Figure 8C). Many enzymes are involved in the biosynthesis pathways of leucine and valine (as shown in Figure 8D). Our results showed that two genes (*BCAT2* and *IPMSA*) in the stems and/or leaves of sweet sorghum showed increasing trends in sweet sorghum after PEG treatment (Figure 8D). In addition, the expressions of genes involved in the biosynthesis pathways of serine (*PGDH2*, *PGDH3*, *SHMT4*, and *PSAT1*) and arginine (*ASS* and *ASL*) were significantly upregulated in the leaves but remained unchanged or significantly downregulated in the stems after PEG treatment (Figure 8E,F). 

### 2.8. The Expression Patterns of Key Genes Involved in the Biosynthesis Pathways of Sugars and Amino Acids under Osmotic Stresses

Finally, we selected five upregulated DEGs (*RAFS2-1*, *GOLS2-1*, *TPS6-2*, *SUS1* and *SUS2*) involved in sugar biosynthesis pathways and three upregulated DEGs (*P5CS2*, *P5CR* and *BCAT2*) involved in amino acid biosynthesis from our transcriptome data, and further investigated the expressions of these genes after 20% PEG treatment for 3–72 h using the qRT-PCR method.

**Figure 9:**
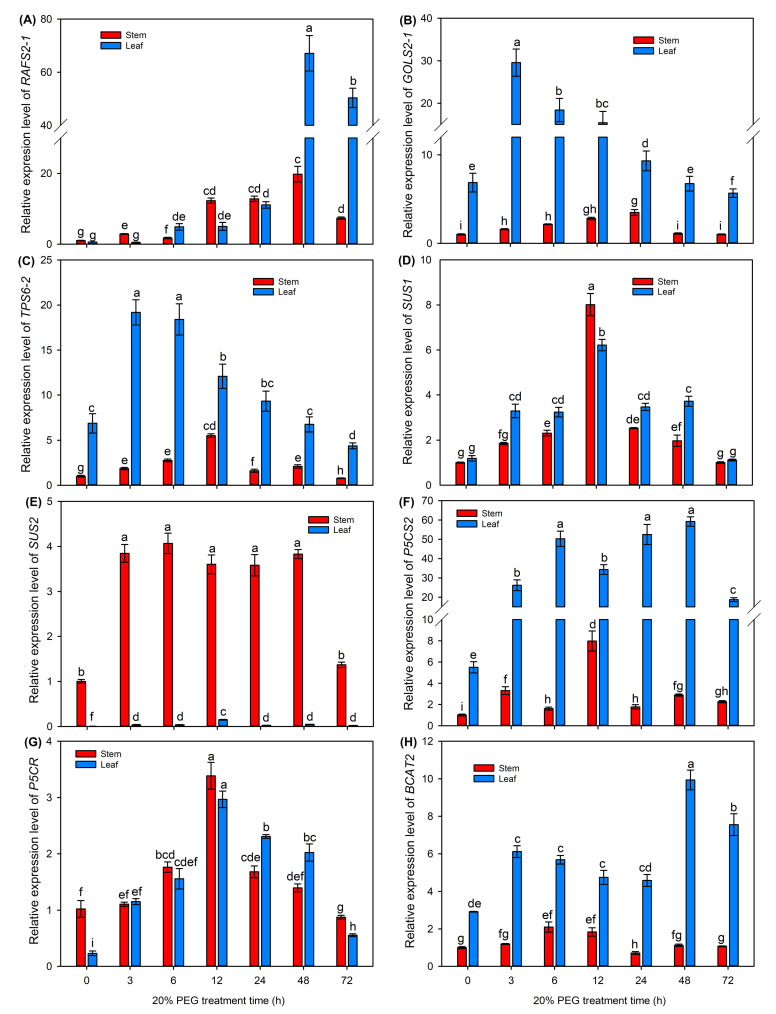
The relative expression levels of key genes involved in soluble sugar and amino acid biosynthesis after 20% PEG treatment for 0–72 h. (**A**) the relative expression level of *RAFS2-1*; (**B**) the relative expression level of *GOLS2-1*; (**C**) the relative expression level of *TPS6-2*; (**D**) the relative expression level of *SUS1*; (**E**) the relative expression level of *SUS2*; (**F**) the relative expression level of *P5CS2*; (**G**) the relative expression level of *P5CR*; (**H**) the relative expression level of *BCAT2*. Data are means (±SDs), *n* = 3. Different letters indicate significant differences as determined by Tukey’s HSD test (*p* < 0.05).

As shown in Figure 9, all the selected genes showed increasing expression trends in the stems and leaves after 20% PEG treatment. Specifically, the expression levels of *RAFS2-1* and *BCAT2* in the leaves were significantly increased and reached peaks under long-term treatment (48 and 72 h, Figure 9A,H), while the expression levels of *GOLS2-1* and *TPS6-2* in the leaves immediately peaked under short-term treatment (3 and 6 h) and then gradually decreased (Figure 9B,C); the expression levels of *SUS2* in the stems and *P5CS2* in the leaves were significantly increased after PEG treatment and continuously maintained at high levels with the prolongation of treatment time (Figure 9E,F); the expression levels of *SUS1* and *P5CR* in both stems and leaves were gradually increased, reached peaks at 12 h and then gradually decreased (Figure 9D,G).

## 3. Discussion

### 3.1. Sweet Sorghum Could Efficiently Enhance the Osmotic Adjustment Ability under Osmotic Stresses

The osmotic adjustment (OA) could effectively lower the water potential of plant tissues by accumulating high amounts of osmolytes to enable water influx into cells, and therefore, play an important role in plants coping with water scarcity [17]. Nevertheless, the OA ability in sweet sorghum under abiotic stresses has not been well studied. In this study, the leaf water potential of sweet sorghum significantly lowered with the increase in external PEG concentrations (Table 1), indicating that the OA ability in sweet sorghum is strengthened under osmotic stresses. Correspondingly, the leaf osmotic potential of PEG-treated seedlings was also significantly decreased (Table 1), suggesting that sweet sorghum could accumulate higher quantities of osmolytes in the leaves when confronted with osmotic stresses. Under normal conditions, K^+^ and NO_3_^−^ are the major inorganic osmolytes for most plant species [41]. As mineral nutrients, the absorption and transport of K^+^ and NO_3_^−^ in plants highly depend on water movement [42]. A previous study found that the NO_3_^−^ content in the leaves of sweet sorghum maintained stable after short-term (2–4 d) osmotic stress [43]. In the present study, we determined the K^+^ and NO_3_^−^ contents in roots, stems, and leaves and found that the contents of both ions in the stems were significantly decreased after 30% PEG treatment for 5 d, while their accumulations in the leaves sustained at high levels (Figure 3). These results suggested that sweet sorghum could transport more K^+^ and NO_3_^−^ from stems into leaves under short-term osmotic stresses. However, it has been reported that the leaf NO_3_^−^ content in sweet sorghum is significantly decreased under long-term osmotic stress [43]. Therefore, the enhanced leaf OA ability in sweet sorghum under osmotic stresses should not mainly rely on the inorganic ions. Instead, our results showed that the accumulations of many organic metabolites in the leaves of sweet sorghum were increased after PEG treatment (Figure 4D and Table S3), indicating that organic metabolites might be the major contributors to leaf OA of sweet sorghum under osmotic stresses.

The maintenance of turgor pressure is essential for cell division and elongation, which plays an important role in the leaf expansion of plants [17]. In this study, the leaf turgor pressure in sweet sorghum was significantly decreased under 20% and 30% PEG treatments (Table 1), which should be a reason why the leaves of seedlings under these treatments were visually thinner than those under the control condition (Figure 1A). On the other hand, the reduced leaf area might contribute to diminishing water loss to enhance the tolerances of sweet sorghum to osmotic stresses.

### 3.2. The Large Accumulation of Soluble Sugars for Osmotic Adjustment Is a Key Strategy of Sweet Sorghum in Adaptation to Osmotic Stresses

Soluble sugars are considered to be indispensable organic osmolytes for plants [14]. Previous studies have found that, under drought stresses, the sucrose content in the stems of sweet sorghum after anthesis is increased significantly, while the content of this sugar in sweet sorghum after physiological maturity is even decreased [6,22]. However, the accumulation change in sucrose in tissues of this species at the vegetative growth stage under conditions of water deficiency has not been reported. Our metabolome data showed that the accumulation of sucrose in the stems and leaves of sweet sorghum seedlings were significantly increased after 20% PEG treatment for 5 d (Figure 5A). Researchers have analyzed the transcriptome of two grain sorghum cultivars RTx430 and BTx642 in the field-drought conditions, and they identified 187 and 766 DEGs in the leaves of 3-week-old seedlings under drought treatment for 1 week [44]. In this study, 1711 and 4978 DEGs in the stems, as well as 2061 and 6596 DEGs in the leaves of sweet sorghum were identified after 20% PEG treatment for 6 and 48 h, respectively (Figure 6E). Several DEGs involved in sucrose metabolic process are detected exclusively in the roots of grain sorghum under drought treatments [44]. Differently, in the stems and leaves of sweet sorghum, the expressions of key genes involved in sucrose biosynthesis such as *SUSs* were highly inducible by PEG treatment, while the expressions of genes involved in the degradation of this sugar such as *INVs* were downregulated after PEG treatment (Figure 7 and Figure 9D,E). Therefore, the increased biosynthesis of sucrose in the shoots should play an important role in the osmotic adjustment of sweet sorghum at the seedling stage under osmotic stresses. In addition, our results also showed that the accumulation of maltose in both stems and leaves of sweet sorghum was increased after PEG treatment (Figure 5A). It is well known that maltose is an essential sugar for alcoholic fermentation [45]. Thus, the cultivation of sweet sorghum in conditions of water deficiency might improve its application value in the alcohol industry.

Except for sucrose and maltose, the accumulations of raffinose, trehalose, D-glucaric acid, gluconolactone, melibiose and alpha-lactose were also significantly increased in the leaves of sweet sorghum after 20% PEG treatment (Figure 5A). The Me value (reflects the relative content of each metabolite) of raffinose (1.71), melibiose (330.48), alpha-lactose (1.12) and trehalose (519.78) was much higher than that of sucrose (0.84) after 20% PEG treatment (Table S3). Considering that the leaf OA ability of sweet sorghum was substantially strengthened under osmotic stresses (Table 1), these soluble sugars are probably essential osmolytes for sweet sorghum as well. Furthermore, the accumulations of raffinose, melibiose and alpha-lactose were also significantly increased in the stems after PEG treatment (Figure 5A), suggesting that osmotic stress could be an inducement for sweet sorghum to accumulate high amounts of sugars in the shoots. In addition, raffinose and trehalose have been proven to also exert protective roles on proteins and membranes in plants [20,21]. In this study, several *TPP* and *TPS* (involved in trehalose biosynthesis), as well as *RAFS* and *GOLS* (involved in raffinose biosynthesis) showed upregulating expression trends in sweet sorghum under PEG treatment (Figure 7 and Figure 9A–C). Thus, raffinose and trehalose should play crucial roles in the adaptation of sweet sorghum to water scarcity.

The starch is a polysaccharide that can be degraded into soluble sugars by amylases [36]. A previous study identified DEGs involved in the starch catabolic process in the leaves of grain sorghum under drought stresses [44]. Similarly, we identified several upregulated DEGs encoding alpha-amylase and beta-amylase in the stems and leaves of sweet sorghum after PEG treatment in this study (Figure 7), suggesting that the degradation of starch is another way from which soluble sugars are generated under osmotic stresses.

### 3.3. Amino Acids Play Crucial Roles in the Response of Sweet Sorghum to Osmotic Stresses

Proline is an important osmolyte and ROS scavenger in plants [46]. In the present study, the accumulation of proline in both stems and leaves of sweet sorghum was significantly increased by over fivefold under 20% PEG treatment (Figure 5B). However, the mechanisms underlying the biosynthesis of proline in sweet sorghum under drought or osmotic stress have not been reported. It has been found that the expression of *P5CS2* (encoding a rate-limiting enzyme for proline biosynthesis) is upregulated in grain sorghum under drought treatments [44]. Similarly, our transcriptome data also showed that the expression of *P5CS2* in the stems and leaves of sweet sorghum was upregulated after 20% PEG treatment for both 6 and 48 h (Figure 8C). The transcripts of *P5CS1* and *P5CR* in the leaves of sweet sorghum were also found to be significantly upregulated after PEG treatment (Figure 8C), suggesting that *P5CS1* and *P5CR* should play indispensable roles in proline biosynthesis in sweet sorghum under conditions of water scarcity. Moreover, qRT-PCR results showed that the expressions of *P5CS2* and *P5CR* in the leaves of sweet sorghum were immediately increased after 20% PEG treatment for 3 h, and the expression of *P5CS2* was continuously sustained at high levels after PEG treatment for 6–72 h (Figure 9F,G). Differently, the expression of *P5CS* in the leaves of barley (*Hordeum vulgare*) is unchanged under drought stresses for 3 days [47]; although the expression of *P5CS* in wheat (*Triticum aestivum*) is significantly increased after drought treatment for 48 h, its expression level decreased to control levels when the treatment time was prolonged to 72 h [48]. Therefore, the rapid increase and continuously high expression of *P5CS* and *P5CR* should be conducive for the large accumulation of proline and, therefore, play crucial roles in sweet sorghum adapting to conditions of water scarcity.

The branched-chain amino acids (BCAAs) including leucine, isoleucine and valine are essential amino acids for humans and animals, but they cannot de novo synthesize these amino acids [26,49]. Therefore, BCAAs in plants are important extracts in industrial productions. In this study, the accumulations of leucine and valine in both stems and leaves of sweet sorghum were found to significantly increase under PEG treatment (Figure 5B), suggesting that the biosynthesis of these two BCAAs in sweet sorghum is enhanced by osmotic stresses. Branched-chain amino acid aminotransferases (BCATs) are key enzymes catalyzing the biosynthesis of all three BCAAs [50]. It has been reported that the expression of *BCATs* in the coleoptiles of barley and in the leaves of durum wheat (*Triticum turgidum*) is induced by drought stresses [51,52]. However, the responses of *BCATs* in sweet sorghum to drought or osmotic stress have not been well-documented so far. Our transcriptome data identified an upregulated transcript of BCAT2 in sweet sorghum after 20% PEG treatment (Figure 8D); qRT-PCR results further confirmed that the expression of *BCAT2* in shoots, especially in the leaves, was drastically induced by PEG treatment (Figure 9H). Therefore, the upregulated expression of *BCAT2* should be closely associated with the large accumulation of BCAAs and as a consequence, it contributes to leaf OA of sweet sorghum. Several members of amino acids are biosynthesized from the same substrate [31]. Thus, enhancing the ability of plants to biosynthesize one specific amino acid might conversely suppress the biosynthesis of other amino acids. Additionally, the substrates for the biosynthesis of three BCAAs in plants are totally different [13]. Therefore, the improvement in the ability to biosynthesize BCAAs should be a promising approach to enhance the drought tolerance of sweet sorghum.

In addition, we found that some other amino acids such as glutamic acid, arginine, 5-aminopentanoic acid, serine, homocystine, argininosuccinic acid, and alanine showed increasing accumulations in stems and/or leaves after PEG treatment (Figure 5B). These amino acids should also play important roles in the adaptation of sweet sorghum to osmotic stresses. Meanwhile, our result showed that the expressions of key genes involved in serine biosynthesis (*PGDH*, *SHMT*, and *PSAT*) and arginine biosynthesis (*ASS* and *ASL*) were only upregulated in the leaves of sweet sorghum after PEG treatment (Figure 8E,F). Interestingly, the accumulations of serine and arginine were significantly increased in the stems under PEG treatment (Figure 5B). Therefore, serine and arginine should be mainly biosynthesized in the leaves of sweet sorghum, after which they are transported into the stems under osmotic stresses.

## 4. Materials and Methods

### 4.1. Plant Material and Growth Conditions

Seeds of a sweet sorghum species, “Lvjure”, were sterilized with 75% ethanol and sown in 0.5 L plastic pots containing coarse silica sand. After germination, seedlings were cultured with modified Hoagland solution (4 mM KNO_3_, 1 mM KH_2_PO_4_, 1 mM MgSO_4_, 1 mM Ca(NO_3_)_2_, 60 μM Fe-citrate, 50 μM H_3_BO_3_, 10 μM MnCl_2_, 1.6 μM ZnSO_4_, 0.6 μM CuSO_4_, and 0.05 μM Na_2_MoO_4_) in a controlled growth chamber with the photoperiod of 16/8 h light/dark at 30/25 °C, light intensity of ~600 μmol·m^−2^·s^−1^ and relative humidity of ~60% [8].

To investigate the effects of osmotic stress on the growth, photosynthesis, ion accumulation and osmotic adjustment ability of sweet sorghum, 3-week-old uniform seedlings grown in silica sand were exposed to Hoagland solution containing 0 (control), 10%, 20% and 30% PEG6000 solutions for 5 d, then tissue samples were harvested. Six replicates were used for each sampling (*n* = 6).

As the physiological analysis showed, 10% PEG treatment had a minimal impact on the growth of sweet sorghum, the 20% PEG treatment began to significantly inhibit seedling growth, and 30% PEG treatment severely impaired seedlings (as presented in the results). To analyze the accumulation changes in soluble sugars and amino acids in sweet sorghum after osmotic stress, 3-week-old uniform seedlings grown in silica sands were irrigated with Hoagland solution (control) or exposed to Hoagland solution containing 20% PEG for 5 d, then the stem and leaf samples were harvested for metabolome profiling. Six replicates were used for each sampling (*n* = 6).

For transcriptome sequencing, 3-week-old uniform seedlings grown in silica sands were likewise irrigated with Hoagland solution (control) or exposed to Hoagland solution containing 20% PEG for 6 and 48 h, respectively, then the stem and leaf samples were collected. Three replicates were used for each sampling (*n* = 3).

To further test the expression patterns of key genes involved in the sugar and amino acid biosynthesis under osmotic stresses using the qRT-PCR method, 3-week-old uniform seedlings grown in silica sands were exposed to Hoagland solution containing 20% PEG for 0, 3, 6, 12, 24, 48 and 72 h, respectively, then the stem and leaf samples were collected. Three replicates were used for each sampling (*n* = 3).

### 4.2. Determination of Growth-Related Parameters

The plant height (PH) and fresh weight (FW) of roots, stems and leaves were determined first. Then, all samples were thoroughly dried in an 80 °C oven to determine the dry weight (DW) of roots, stems and leaves. Finally, the leaf relative water content (RWC) was calculated as (FW − DW)/DW [17].

Fresh leaf samples were crushed thoroughly with 10% trichloroacetic acid and mixed with 0.6% thiobarbituric acid at 100 °C to extract the malondialdehyde (MDA). After centrifuging, the absorbances at 532 and 600 nm in the supernatant were measured using a UV spectrophotometer (UV-2102C, Unico Instrument Co., Ltd., Shanghai, China) to determine the MDA content [53].

### 4.3. Determination of Photosynthesis-Related Parameters

The LI-6800 Photosynthesis System (LI-COR Biosciences, Lincoln, NE, USA) was used to measure the leaf gas-exchange indexes, including net photosynthesis rate (Pn), stomatal conductance (Gs), intercellular CO_2_ concentration (Ci) and transpiration rate (Tr). The measurements were conducted in the growth chamber between 3 h and 6 h after the start of the photoperiod. The leaf water-use efficiency (WUE) was calculated as Pn/Gs [17].

Fresh leaf samples were immersed in a mixed solution of 80% acetone and 95% ethanol (1:1, *v*/*v*) at 4 °C in the dark to extract the chlorophyll. After centrifuging, the absorbances at 645 and 663 nm in the supernatant were measured to calculate the chlorophyll content [54].

### 4.4. Determination of Tissue K^+^ and NO_3_^−^ Contents

As K^+^ and NO_3_^−^ are two members of major inorganic osmolytes in plants [41], we determined the contents of these ions in the roots, stems, and leaves. Briefly, the K^+^ in the oven-dried root, stem, and leaf samples was extracted with 100 mM glacial acetic acid at 100 °C, and then the Model 410 flame spectrophotometer (Sherwood Scientific, Ltd., Cambridge, UK) was employed to determine the K^+^ content [17]. NO_3_^−^ in oven-dried tissue samples was extracted with a 5% salicylic acid-H_2_SO_4_ mixture, and then the NO_3_^−^ content was determined using the colorimetric method with salicylic acid [55].

### 4.5. Determination of Leaf Osmotic Adjustment-Related Parameters

A water potential system (C-52, Wescor, UT, USA) was used to determine leaf water potential (Ψ_w_). Briefly, the leaf blade was settled in a pressure chamber with the cut noodle exposed to air; then, the pressure in the chamber was gradually increased using a pump. When the water drops emerged at cut noodle, the value of the pressure in the chamber was immediately recorded to reflect the leaf Ψ_w_. The leaf samples were transiently frozen in liquid nitrogen; after thawing, the saps in the leaves were collected, and the osmolality concentration in the leaf saps was analyzed using the Osmomat-070 cryoscopic osmometer (Gonotec GmbH, Berlin, Germany) to determine osmotic potential (Ψ_S_) [56]. The turgor pressure (Ψ_t_) = Ψ_w_ − Ψ_S_ [57].

### 4.6. Metabolome Analysis

The freeze-dried stem and leaf samples were crushed into powder, then mixed with extraction solution (MeOH:ACN:H_2_O, 2:2:1). LC-MS/MS analyses were performed by Wuhan Benagen Technology Co., Ltd. (Wuhan, China) using an UHPLC system (Vanquish, Thermo Fisher Scientific, Waltham, MA, USA). Briefly, the metabolites in the above liquid mixtures were separated with a liquid chromatography column (Kinetex C18, Phenomenex, Torrance, CA, USA); then the Orbitrap Exploris 120 mass spectrometer (Thermo Fisher Scientific, Waltham, MA, USA) was used for detecting MS/MS spectra. The raw data were processed for peak detection, extraction, alignment and integration using a XCMS-based R package, and a self-built secondary mass spectrometry database (BiotreeDB, V2.1) was applied in metabolite annotation.

To analyze the accumulation changes in metabolites after PEG treatment, the abundance of each metabolite in the treatment group against the corresponding control group was performed, and the differentially accumulated metabolites (DAMs) were screened out by using thresholds of VIP ≥ 1, |log_2_fold change| ≥ 1, and *p* < 0.05 [26].

### 4.7. Transcriptome Sequencing

Total RNA in stem and leaf samples was extracted and the first- and second-strand cDNA were synthesized [58]. cDNA samples were then used for RNA sequencing at BGI platform (Beijing Genomics Institute, Shenzhen, China).

The FPKM values of transcripts in each library were calculated using RSEM software (V1.3.3). Then, gene expression changes were performed using DESeq2 software (V1.26.0). Finally, differentially expressed genes (DEGs) were screened out by using thresholds of |log_2_fold change| ≥ 1 and *p* < 0.05 [8].

The relative expression levels of 20 randomly selected DEGs were determined by the qRT-PCR method to validate the reliability of transcriptome data. A real-time PCR Thermocycler (LightCycler 480 System, Roche, Basel, Switzerland) was employed, and the cDNA samples synthesized in transcriptome sequencing were used as templates. *SbEIF4α* was used as an endogenous control gene [59]. Finally, the correlation analysis was performed [8].

### 4.8. qRT-PCR Analysis

We selected five DEGs involved in sugar biosynthesis (*RAFS2-1*, *GOLS2-1*, *TPS6-2*, *SUS1* and *SUS2*) and three DEGs involved in amino acid biosynthesis (*P5CS2*, *P5CR* and *BCAT2*) to further analyze the expression patterns of these genes under PEG treatment for 0–72 h using the qRT-PCR method. The total RNA in stem and leaf samples was extracted from the Trizol reagent (Tiangen, Beijing, China). After the removal of genomic DNA, RNA samples were converted into cDNA using the PrimeScript™ RT Master Mix (Takara, Dalian, China). The cDNA samples were used as templates for qRT-PCR with the LightCycler 480 System [8].

### 4.9. Data Analysis

Six replicates were used for physiological parameter measurements and metabolome profiling (*n* = 6), three replicates were used for transcriptome sequencing and qRT-PCR analysis (*n* = 3). The data were subjected to one-way analysis of variance (ANOVA) using SPSS19.0 (IMB Corp, Armonk, NY, USA) followed by Tukey’s HSD to detect significant differences (*p* < 0.05).

## 5. Conclusions

Sweet sorghum exhibits a substantially enhanced osmotic adjustment ability to maintain leaf water hydration status under osmotic stresses; however, the contents of inorganic ions, including K^+^ and NO_3_^−^ in the leaves, remain stable. Differently, soluble sugars such as sucrose, trehalose, and raffinose, as well as amino acids such as proline, leucine, valine, and arginine, should play essential roles in the adaptation of sweet sorghum to water scarcity, as their accumulations in stems and leaves are significantly increased under osmotic stresses, which are possibly attributed to the upregulated expressions of key genes such as *SUS*, *TPS*, *RAFS*, *GOLS*, *P5CS*, *P5CR*, and *BCAT* involved in the biosynthesis pathways of these metabolites. All these results provide a theoretical basis for the large-scale cultivation of sweet sorghum in arid regions, and further study on the molecular basis underlying biosynthesis mechanisms of soluble sugars and amino acids would help to uncover the adaptative mechanisms of this species to water scarcity.

## Figures and Tables

**Table 1:** Effects of 10–30% PEG treatments on the leaf water potential, osmotic potential, and turgor pressure of sweet sorghum.

PEG Concentrations	Water Potential (Ψ_w_, MPa)	Osmotic Potential (Ψ_s_, MPa)	Turgor Pressure (Ψ_t_, MPa)
0	−0.20 ± 0.01 a	−0.64 ± 0.02 a	0.44 ± 0.02 a
10%	−0.52 ± 0.02 b	−0.90 ± 0.04 b	0.38 ± 0.02 a
20%	−0.67 ± 0.02 c	−0.98 ± 0.03 c	0.31 ± 0.02 b
30%	−1.17 ± 0.05 d	−1.36 ± 0.04 d	0.19 ± 0.01 c

Data are means (±SDs), *n* = 6. Different letters indicate significant differences determined by Tukey’s HSD test (*p* < 0.05).

**Table 2:** The statistics of the transcriptome sequencing data of sweet sorghum after 20% PEG treatment for 6 and 48 h.

Samples	Raw Reads	Raw Bases	Clean Reads	Clean Bases	Q20 Rate	Q30 Rate	GC Content
C6S-1	70,005,740	10,500,861,000	70,001,138	10,075,876,352	98.28%	94.51%	56.69%
C6S-2	70,053,232	10,507,984,800	70,049,684	10,089,966,374	98.37%	94.77%	55.03%
C6S-3	70,007,322	10,501,098,300	70,003,242	10,048,450,864	98.33%	94.67%	57.02%
C6L-1	70,056,834	10,508,525,100	70,053,032	10,093,545,900	98.58%	95.41%	58.91%
C6L-2	70,045,804	10,506,870,600	70,041,984	10,064,095,400	98.35%	94.73%	58.03%
C6L-3	70,001,696	10,500,254,400	69,997,174	10,145,080,392	98.32%	94.60%	57.59%
O6S-1	70,040,106	10,506,015,900	70,035,658	10,057,149,930	98.48%	95.15%	57.09%
O6S-2	70,056,348	10,508,452,200	70,051,780	10,105,534,410	98.35%	94.74%	57.66%
O6S-3	70,044,758	10,506,713,700	70,040,118	10,020,534,800	98.26%	94.46%	56.76%
O6L-1	70,017,270	10,502,590,500	70,013,280	10,101,772,052	98.43%	94.97%	58.11%
O6L-2	70,022,412	10,503,361,800	70,018,022	10,120,521,096	98.36%	94.77%	56.89%
O6L-3	70,060,316	10,509,047,400	70,056,164	10,164,256,440	98.38%	94.79%	57.55%
C48S-1	70,033,840	10,505,076,000	70,030,128	10,071,890,906	98.37%	94.74%	57.69%
C48S-2	70,041,786	10,506,267,900	70,037,872	10,084,509,768	98.35%	94.72%	56.84%
C48S-3	70,049,066	10,507,359,900	70,045,014	10,004,322,094	98.32%	94.63%	56.42%
C48L-1	70,035,786	10,505,367,900	70,032,108	10,168,334,388	98.31%	94.59%	57.13%
C48L-2	70,001,862	10,500,279,300	69,998,332	10,099,317,072	98.37%	94.76%	57.74%
C48L-3	70,021,386	10,503,207,900	70,017,298	10,119,140,744	98.30%	94.58%	56.92%
O48S-1	70,061,314	10,509,197,100	70,057,064	10,074,893,538	98.44%	94.97%	55.84%
O48S-2	70,053,770	10,508,065,500	70,049,636	10,042,732,892	98.39%	94.82%	56.13%
O48S-3	70,044,590	10,506,688,500	70,040,532	10,086,926,960	98.56%	95.38%	56.64%
O48L-1	70,009,878	10,501,481,700	70,006,172	10,156,806,710	98.31%	94.58%	57.14%
O48L-2	70,029,000	10,504,350,000	70,024,454	10,138,265,670	98.42%	94.93%	55.70%
O48L-3	70,009,212	10,501,381,800	70,005,354	10,062,381,550	98.59%	95.45%	56.32%

**Note:** C6S represents the stem samples irrigated with Hoagland solution for 6 h, C6L represents the leaf samples irrigated with Hoagland solution for 6 h, O6S represents the stem samples treated with 20% PEG for 6 h, O6L represents the leaf samples treated with 20% PEG for 6 h, C48S represents the stem samples irrigated with Hoagland solution for 48 h, C48L represents the leaf samples irrigated with Hoagland solution for 48 h, O48S represents the stem samples treated with 20% PEG for 48 h, O48L represents the leaf samples treated with 20 PEG for 48 h. Each sampling had three replicates (*n* = 3).

## Data Availability

The transcriptome data of all samples have been uploaded to the NCBI Sequencing Read Archive (SRA) database (https://www.ncbi.nlm.nih.gov) under the accession number PRJNA1102863. The data that support the findings of this study are available from the corresponding author upon reasonable request.

## Supplementary Materials

The following supporting information can be downloaded at: https://www.mdpi.com/article/10.3390/ijms25168942/s1.
