# Supplementary material for: Physiological, Metabolome and Gene Expression Analyses Reveal the Accumulation and Biosynthesis Pathways of Soluble Sugars and Amino Acids in Sweet Sorghum under Osmotic Stresses
Source: Int J Mol Sci. 2024 Aug 16;25(16):8942. doi: 10.3390/ijms25168942 (PMC11354453; doi:10.3390/ijms25168942)
Supplement: Supplementary file 1 [file ijms-25-08942-s001.zip › Supplementary Figures S1 and S2.pdf]

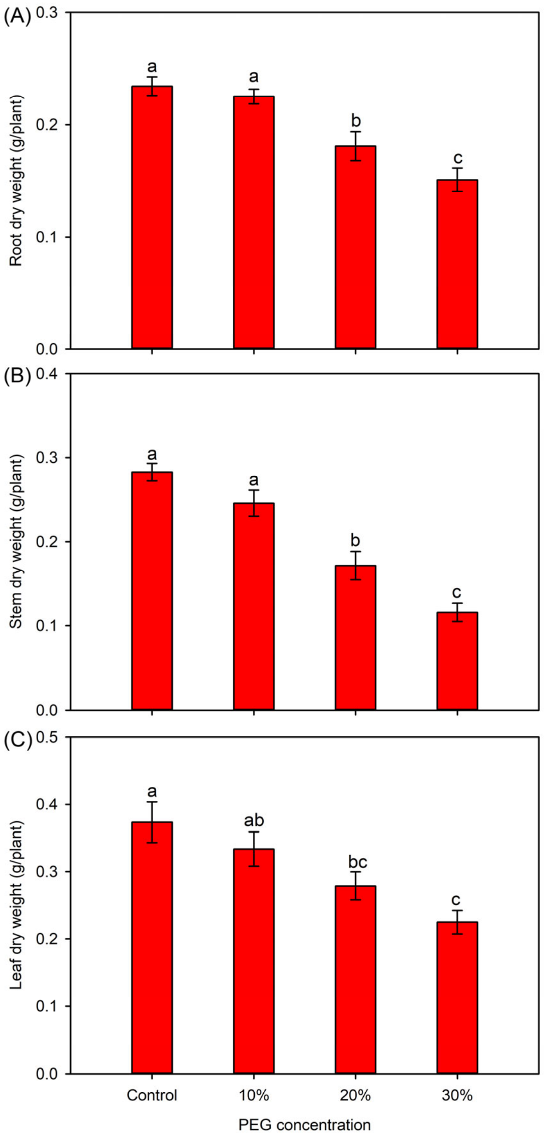

Figure S1. Effects of 10%–30% PEG treatments on the tissue dry weight of sweet sorghum. (**A**) Root dry weight; (**B**) stem dry weight and (**C**) leaf dry weight. Data are means (±SD), $n = 6$. Different letters indicate significant differences as determined using Tukey's HSD test ($p < 0.05$).

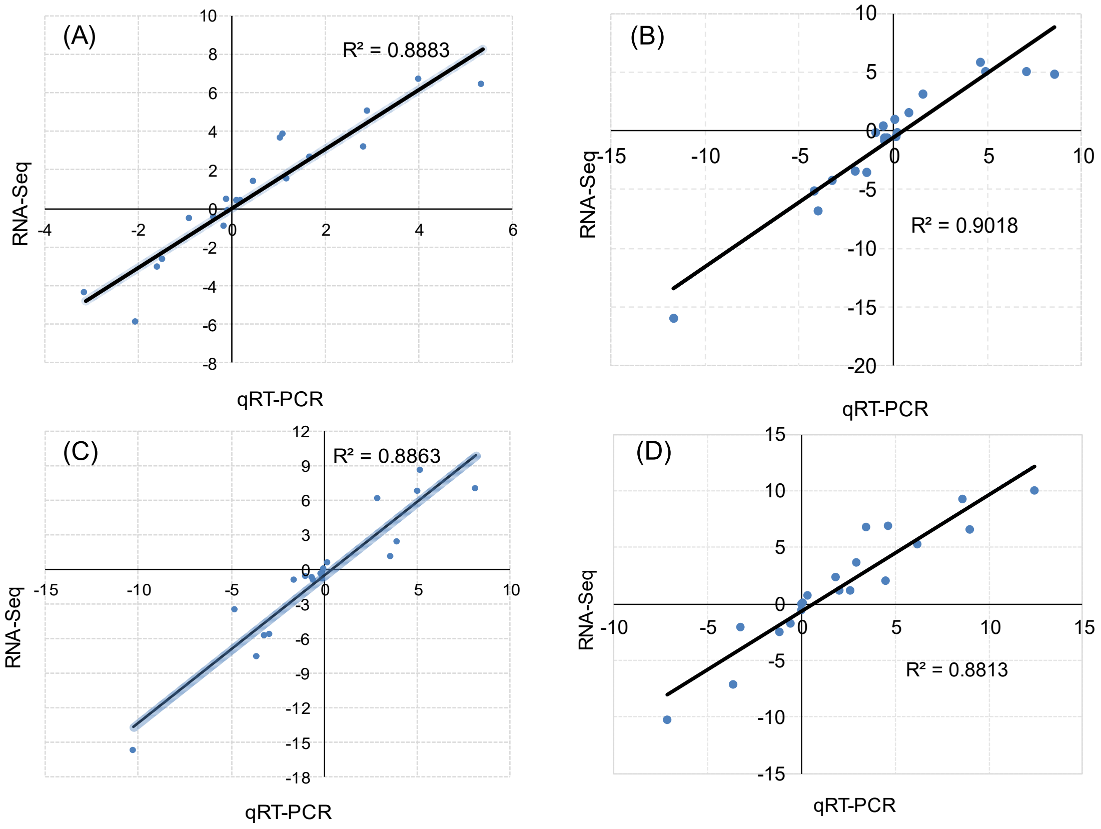

Figure S2. Correlation analysis for expression pattern validation of 20 randomly selected DEGs under 20% PEG treatment for 6 h and 48 h in stems ((**A**) and (**C**), respectively) and leaves ((**B**) and (**D**), respectively). The X-axes and Y-axes show the gene transcript level changes obtained by RNA-seq and qRT-PCR, respectively. R^2 indicates the correlation.
